# Evidence of Stage Shift in Women Diagnosed With Ovarian Cancer During Phase II of the United Kingdom Familial Ovarian Cancer Screening Study

**DOI:** 10.1200/JCO.2016.69.9330

**Published:** 2017-02-27

**Authors:** Adam N. Rosenthal, Lindsay S.M. Fraser, Susan Philpott, Ranjit Manchanda, Matthew Burnell, Philip Badman, Richard Hadwin, Ivana Rizzuto, Elizabeth Benjamin, Naveena Singh, D. Gareth Evans, Diana M. Eccles, Andy Ryan, Robert Liston, Anne Dawnay, Jeremy Ford, Richard Gunu, James Mackay, Steven J. Skates, Usha Menon, Ian J. Jacobs, on behalf of the United Kingdom Familial Ovarian Cancer Screening Study collaborators

**Affiliations:** Adam N. Rosenthal, Lindsay S.M. Fraser, Susan Philpott, Ranjit Manchanda, Matthew Burnell, Philip Badman, Richard Hadwin, Ivana Rizzuto, Andy Ryan, Robert Liston, Jeremy Ford, Richard Gunu, Usha Menon, and Ian J. Jacobs, University College London Elizabeth Garrett Anderson Institute for Women’s Health; Elizabeth Benjamin, University College London; Naveena Singh, Barts Health National Health Service Trust; Ranjit Manchanda, Barts Cancer Institute, Queen Mary University of London; Anne Dawnay, University College London Hospital; James Mackay, The University College London Cancer Institute, London; D. Gareth Evans, University of Manchester, St Mary’s Hospital Manchester, Manchester; Diana M. Eccles, Southampton General Hospital, Southampton, United Kingdom; Steven J. Skates, Massachusetts General Hospital and Harvard Medical School, Boston, MA; and Ian J. Jacobs, University of New South Wales Australia, Sydney, New South Wales, Australia.

## Abstract

**Purpose:**

To establish the performance of screening with serum cancer antigen 125 (CA-125), interpreted using the risk of ovarian cancer algorithm (ROCA), and transvaginal sonography (TVS) for women at high risk of ovarian cancer (OC) or fallopian tube cancer (FTC).

**Patients and Methods:**

Women whose estimated lifetime risk of OC/FTC was ≥ 10% were recruited at 42 centers in the United Kingdom and underwent ROCA screening every 4 months. TVS occurred annually if ROCA results were normal or within 2 months of an abnormal ROCA result. Risk-reducing salpingo-oophorectomy (RRSO) was encouraged throughout the study. Participants were observed via cancer registries, questionnaires, and notification by centers. Performance was calculated after censoring 365 days after prior screen, with modeling of occult cancers detected at RRSO.

**Results:**

Between June 14, 2007, and May 15, 2012, 4,348 women underwent 13,728 women-years of screening. The median follow-up time was 4.8 years. Nineteen patients were diagnosed with invasive OC/FTC within 1 year of prior screening (13 diagnoses were screen-detected and six were occult at RRSO). No symptomatic interval cancers occurred. Ten (52.6%) of the total 19 diagnoses were stage I to II OC/FTC (CI, 28.9% to 75.6%). Of the 13 screen-detected cancers, five (38.5%) were stage I to II (CI, 13.9% to 68.4%). Of the six occult cancers, five (83.3%) were stage I to II (CI, 35.9% to 99.6%). Modeled sensitivity, positive predictive value, and negative predictive value for OC/FTC detection within 1 year were 94.7% (CI, 74.0% to 99.9%), 10.8% (6.5% to 16.5%), and 100% (CI, 100% to 100%), respectively. Seven (36.8%) of the 19 cancers diagnosed < 1 year after prior screen were stage IIIb to IV (CI, 16.3% to 61.6%) compared with 17 (94.4%) of 18 cancers diagnosed > 1 year after screening ended (CI, 72.7% to 99.9%; *P* < .001). Eighteen (94.8%) of 19 cancers diagnosed < 1 year after prior screen had zero residual disease (with lower surgical complexity, *P* = .16) (CI, 74.0% to 99.9%) compared with 13 (72.2%) of 18 cancers subsequently diagnosed (CI, 46.5% to 90.3%; *P* = .09).

**Conclusion:**

ROCA-based screening is an option for women at high risk of OC/FTC who defer or decline RRSO, given its high sensitivity and significant stage shift. However, it remains unknown whether this strategy would improve survival in screened high-risk women.

## INTRODUCTION

Inherited mutations in *BRCA1* and *BRCA2* and Lynch syndrome (LS) account for a significant minority (15% to 25%) of ovarian cancers (OCs)^1,2^ and confer a high risk for OC: 11% to 37% by age 70 years in *BRCA2* carriers and 39% to 65% in *BRCA1* carriers.^3,4^ Other lower-penetrance homologous repair pathway genes have been implicated in familial OC.^5,6^

Although medium-term survival with *BRCA*-associated OC exceeds that of sporadic OC,^7,8^ the long-term outlook remains poor.^9^ Risk-reducing salpingo-oophorectomy (RRSO) for women older than 35 years of age to prevent OC or fallopian tube cancer (FTC) and to detect occult neoplasia is recommended as the only proven mortality-reducing intervention.^10,11^ Although effective when used premenopausally,^10,11^ RRSO causes infertility and premature menopause, with associated cardiovascular risks,^12^ osteoporosis,^13^ and neurologic risks^14^ (although premature menopause can be treated with hormone replacement therapy). Some women decline RRSO regardless of OC risk, and others prefer to defer it (eg, until menopause). Effective OC screening would be a welcome option for such women.

Annual OC screening in the general population that uses a cutoff for the serum tumor marker cancer antigen 125 (CA-125) was associated with improved survival.^15^ In the high-risk population, we^16^ and others^17-20^ have reported annual screening using a CA-125 cutoff and transvaginal sonography (TVS). Although we demonstrated high sensitivity (> 80%) and positive predictive value (PPV; 25%), two symptomatic interval cancers occurred, and 69% of detected cancers were stage III to IV.^16^ This annual screening interval has been associated with a poor 10-year survival rate of 36% in *BRCA1/2* carriers.^21^

Multimodal screening with the risk of ovarian cancer algorithm (ROCA) to interpret serial CA-125 results, and TVS as a second-line test, in the randomized general-population United Kingdom Collaborative Trial of Ovarian Cancer Screening (UKCTOCS) achieved high sensitivity and specificity.^22,23^ Significantly more (40%) low-volume (stages I, II, or IIIa) invasive epithelial ovarian/peritoneal cancers were identified compared with unscreened controls (26%) on an intention-to-screen analysis, and the trial provided an encouraging, though not definitive, mortality benefit.^24^

Random assignment to a nonscreening arm is thought to be unacceptable to high-risk women and clinicians.^16^ Even if ethical, it would likely be unfeasible, so research screening in this population is limited to prospective cohort studies. To our knowledge, this is the first published study to use ROCA-based screening to define sensitivity in the high-risk population.

## PATIENTS AND METHODS

A prospective multicenter cohort screening study was undertaken within the United Kingdom (UK) National Health Service (NHS). Ethical approval was given by the Eastern Multicentre Research Ethics Committee (Reference No. 97/5/007). The protocol can be found online.^25^

### Entry Criteria

High-risk women at an estimated minimum 10% lifetime risk of OC were recruited; inclusion criteria (Data Supplement, online only) depended on family history or predisposing mutations. Documentation (death certificates and/or histopathology reports) of relevant cancers was required, and eligibility was confirmed by the coordinating center (CC). Clinical genetic testing was performed by accredited NHS laboratories. After screening ended, 45.2% of the study population underwent *BRCA1/2* next-generation sequencing research testing.^26^

### Recruitment

Participants were recruited at 42 centers in the UK by specialist nurses, clinical geneticists, or gynecologists. In December 2006, participants in the UK Familial Ovarian Cancer Screening Study (UK FOCSS) Phase I (annual screening using a CA-125 cutoff and TVS)^16^ were invited to join this study—UK FOCSS Phase II. Other participants were recruited de novo. Women were counseled about RRSO and screening limitations. All participants provided written consent.

### Screening

The trial database^16^ scheduled serum CA-125 tests every 4 months and TVS annually. Venipuncture packs were mailed to participants for use in primary care and blood samples returned to the CC laboratory for CA-125 assay.^23^ Results were uploaded to the database, which calculated OC risk using the high-risk ROCA, which also incorporated the higher a priori risk in our population and different reference levels for risk stratification for postmenopausal compared with premenopausal women, because of the higher baseline CA-125 and variability in premenopausal women.^27^ Menopausal status was determined by the database by using the age of participants and their responses to questions about gynecologic history and/or symptoms, which were returned with serum samples (Data Supplement).

Initial risk of ovarian cancer (ROC) was based on initial CA-125 level and estimated age-specific OC incidence. Subsequently, ROC was based on absolute CA-125 level and rate of change. Initially high or increasing CA-125 levels (even < 30 iU/ml) generated a high ROC, whereas initially low, stable-high (even > 30 iU/ml), or decreasing levels generated low ROCs. ROCA results were used for triage, including expedition of repeat CA-125 tests and/or TVS after abnormal results (Data Supplement).

Collaborating centers performed scans and completed proformas (Data Supplement), which were classified by the database according to predetermined criteria (Data Supplement).^16^ When indicated, women were referred to a gynecologist for clinical assessment, with a view to surgical removal of the fallopian tubes and ovaries. The final decision about surgery was made after additional investigation and discussion with the patient.

### Follow-Up

Participants were flagged (by their unique NHS number) with relevant cancer registries, which provided cancer and/or death data.^16^ Collaborators notified the CC when women withdrew before routine screening ended (June 30, 2011). Women were observed through cancer registries with censorship that was based on date of death, last notification from the registry, or last contact if they were lost to registry follow-up. Participants were sent health questionnaires in January 2011 and April 2013 specifically asking about surgery that involved removal of fallopian tubes/ovaries and cancer diagnosis.

### Diagnostic Documentation

Whenever women underwent salpingo-oophorectomy, the CC obtained documentation of indication, operation notes, and histopathology/cytopathology reports. These were reviewed by a gynecologic oncologist (A.N.R.) and pathologist (E.B./N.S.) and were classified according to the International Statistical Classification of Diseases and Related Health Problems, 10th revision (ICD-10). Trial surgery was defined as either screen-positive or screen-related (nonconcerning abnormal results, such as simple cysts and/or transient/stable abnormal ROC results that contributed to the participant’s decision to undergo surgery).^15^ Centers were provided with an RRSO protocol, which advocated serial sectioning of fallopian tubes/ovaries (Data Supplement). A surgical complexity score was assigned using recognized criteria (Data Supplement).^28^

### Statistical Analysis

For performance analyses, data were censored 365 days after the last UK FOCSS screen. Invasive OC, FTC, or primary peritoneal cancer (PPC) diagnosed < 365 days after the last screen were included. Cancers that occurred after censoring and diagnosed before February 28, 2016 were reported but not included in the performance analyses. The study was powered to estimate sensitivity within 10% (expected 95% CI), given an annual OC incidence of 0.5%. Analyses were done with Stata (version 14; STATA, College Station, TX).

Compliance with blood tests and scans was defined as the proportion of requested tests received by the CC. These were analyzed separately and according to screen type (eg, routine, protocol-indicated repeat).

Women who underwent salpingo-oophorectomy were only classified as having undergone RRSO if they were asymptomatic, they had normal results at prior screen, and the recruiting center indicated RRSO as the reason for withdrawal. Cases in which abnormal results prompted surgery were true positive (TP) if invasive epithelial OC/FTC was diagnosed. All other diagnoses (including borderline/benign tumors) that resulted from surgery that was prompted by abnormal results were false positive (FP). Cases in which nonconcerning test results (simple cysts/transiently elevated CA-125) contributed to the decision for surgery were classified as screen-related surgery, to provide estimates of likely additional surgeries in any future screening program. True-negative (TN) designations were for those patients in whom the last screen was normal and no OC/FTC was diagnosed < 365 days. Patients who presented with clinically diagnosed interval cancers between screens or < 365 days after the final screen were considered false negative (FN). Prevalent cases were those diagnosed at first screen. Incident cases were those diagnosed subsequently. For women who transferred from Phase I (annual CA-125 cutoff and scan) to Phase II (ROCA every 4 months and annual TVS), their first Phase II screen was classified as incident.

We reported performance according to whether occult cancers diagnosed < 365 days after a prior screen were classified as FN or TP.^16^ In an attempt to estimate true sensitivity, we assumed that the proportion of occult cancers identified at RRSO, which would have been screen detected had women not undergone surgery, would be identical to that observed in those who continued screening. We then used the lower confidence limit of observed sensitivity in women who did not undergo RRSO as a conservative estimate of occult cancer detection sensitivity, and we rounded the predicted number of occult cancers detected to the nearest integer.

Because the protocol required parallel CA-125 and TVS, the results of which influenced each other’s timing, it was not possible to calculate performance characteristics per test. Therefore, we calculated these metrics per woman-screen year (WSY) for the protocol overall.

To allocate WSYs to correct outcomes we applied the following rules; for TP and FP detection screens, the WSY that commenced with that screen was classified as TP or FP, respectively. WSYs before the detection screen were TN. For occult cancers diagnosed < 365 days after prior screen, the WSY that commenced with that screen was classified as FN or TP (dependent on analysis type), and prior WSYs were TN. For TN cases, all WSYs were classified TN.

To investigate potentially avoidable delays, we analyzed screening and screen-to-surgery intervals.^16^ Detection screens were defined as an abnormal TVS and/or abnormal ROC that led to a surgery/biopsy that diagnosed OC/FTC. Delayed screens were defined as any detection screen performed after the protocol-indicated date. Delay was calculated as the detection screen date minus the protocol-indicated date. The interval from screen date to diagnosis was calculated to the date of surgery/biopsy. We compared International Federation of Gynecology and Obstetrics stage and postsurgery zero residual disease rates in OC/FTC diagnosed during and < 365 days from the end of UK FOCSS screening with those diagnosed > 365 days after screening ended. We also compared stage-distribution and zero residual disease rates in incident screen-detected cancers in Phases I and II of the study. No survival analysis was performed because of the low number of events observed.

## RESULTS

**Fig 1.**
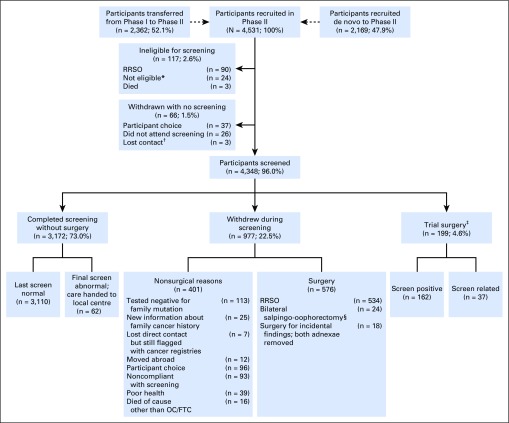
CONSORT diagram. Percentages refer to the proportion of the total in preceding box. (*)Ineligible due to new information about family cancer history, tested negative for family mutation, or already undergoing investigation for abnormal screening results during UK Familial Ovarian Cancer Screening Study Phase I. (†)Unable to establish current whereabouts or nonresponder despite correct address. (‡)Defined as either screen-positive or screen-related (nonconcerning abnormal results, such as simple cysts and/or transient/stable abnormal ROC results that contributed to the participant’s decision to undergo surgery). Includes volunteers who underwent unilateral salpingo-oophorectomy or diagnostic laparoscopy only who returned to screening. (§)Insufficient data to determine indication (all had normal final screen results, none had cancer). RRSO, risk-reducing salpingo-oophorectomy.

Between June 14, 2007, and May 15, 2012, 4,531 women were recruited. This included 2,362 (66.3%) of 3,563 eligible women from UK FOCSS Phase I (Fig 1). Table 1 lists inclusion indications. A total of 183 (4.0%) women withdrew before screening (Fig 1). The outcome of the remaining 4,348 women (96.0%) were analyzed. The median age at recruitment was 45.5 years (range, 34.2 to 84.8 years). Of the eligible women, 1,278 women (29.4% of participants) underwent mutation testing, and 1,965 (45.2%) subsequently underwent next-generation sequencing.^24^ Overall, 924 (21.3%) women were known mutation carriers (further demographics in Data Supplement).

**Table 1.**
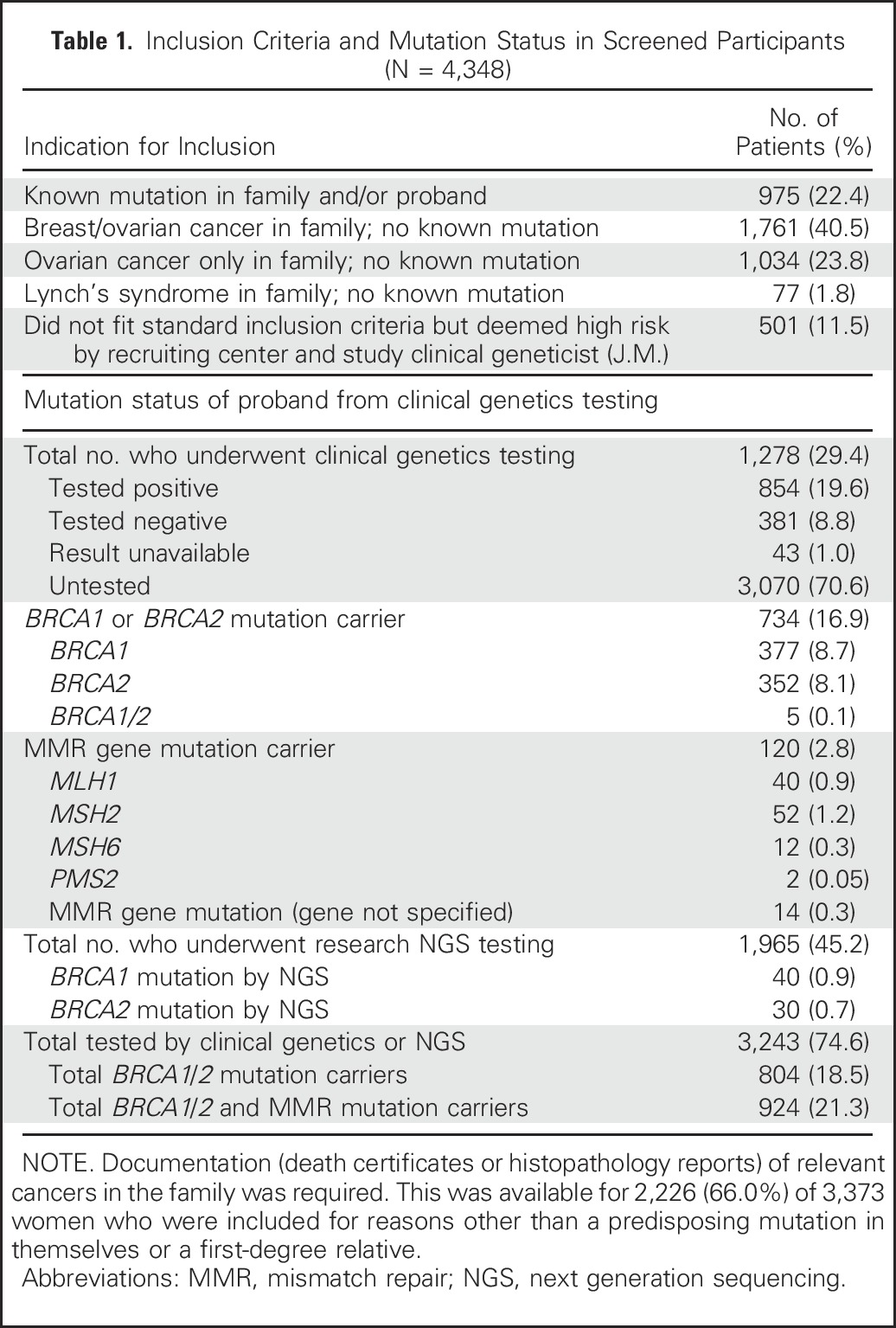
Inclusion Criteria and Mutation Status in Screened Participants (N = 4,348)

The last cancer notifications from NHS Digital were received on February 28, 2016 (England/Wales), May 15, 2016 (Scotland), and April 19, 2016 (Northern Ireland); the last death notifications were received on March 14, 2016 (all countries). Follow-up was possible for 4,046 women (93.1%). Median follow-up beyond last screen/withdrawal was 4.7 years (range, 0 to 8.7 years).

### Screening/Compliance

The 4,348 screened participants underwent 13,728 WSY (median, 3.26 screen-years per woman; range, 1.00 to 5.94 screen-years per woman). A total of 189 women (4.3%) ceased screening by choice. Five hundred fifty-eight (12.8%) ceased because of surgical removal of both fallopian tubes/ovaries for RRSO (n = 534) or indeterminate reasons (n = 24). A total of 377 women (8.7%) whose last screen was abnormal continued screening until May 15, 2012, by which time 315 had normal results and did not undergo surgery. Care was transferred to local gynecologists for the 62 women who still had abnormal results. Three of these 62 women underwent surgery; none had cancer.

Compliance with requested routine CA-125 tests and scans were 92.1% (27,138 of 29,450 CA-125 tests) and 94.6% (9,100 of 9,619 scans), respectively (Data Supplement). Compliance for scans was based on reports received, not scans undertaken, so it is likely an underestimate. Protocol-indicated repeat test compliance was higher: 97.4% (4,716 of 4,834) of blood tests, and 98.8% (2,792 of 2,825 ) of scans requested were received.

Of the 32,587 blood samples received, routine tests comprised 83.3% (27,138 of 32,587), protocol-triggered repeats comprised 14.5% (4,716 of 32,587), and 2.2% (733 of 32,587) were requested by study clinicians (eg, because CA-125 levels had increased by ≥ 50%, despite a normal ROC). A total of 2,233 (6.9%) of 32,587 blood samples were discarded because they arrived more than 56 hours after venipuncture. Of the 12,038 scan results, 75.6% (9,100 of 12,038) were annual, 23.2% (2,792 of 12,038) were triggered early by abnormal ROC results and/or previous abnormal scans, and 1.2% (146 of 12,038) were repeated because of a poor view of the ovaries.

Overall, 162 (3.7%) of 4,348 women underwent screen-positive trial surgery. Thirteen of these 162 women had screen-detected cancers. The remaining 149 (3.4%) of the 4,348 women underwent false-positive surgery prompted by abnormal results (Table 2). Of these 149 women who underwent false-positive surgery, 46 (30.9%) had an abnormal ROC alone, 62 (41.6%) had an abnormal scan alone, and 41 (27.5%) had abnormal results for both tests. Overall, 95 (63.8%) of the 149 women who underwent false-positive surgery had benign ovarian pathology, two (1.3%) had borderline ovarian tumors, and 52 (35.0%) had other/no pathology identified. An additional 37 (0.9%) of the 4,348 women underwent screen-related trial surgery.

**Table 2.**
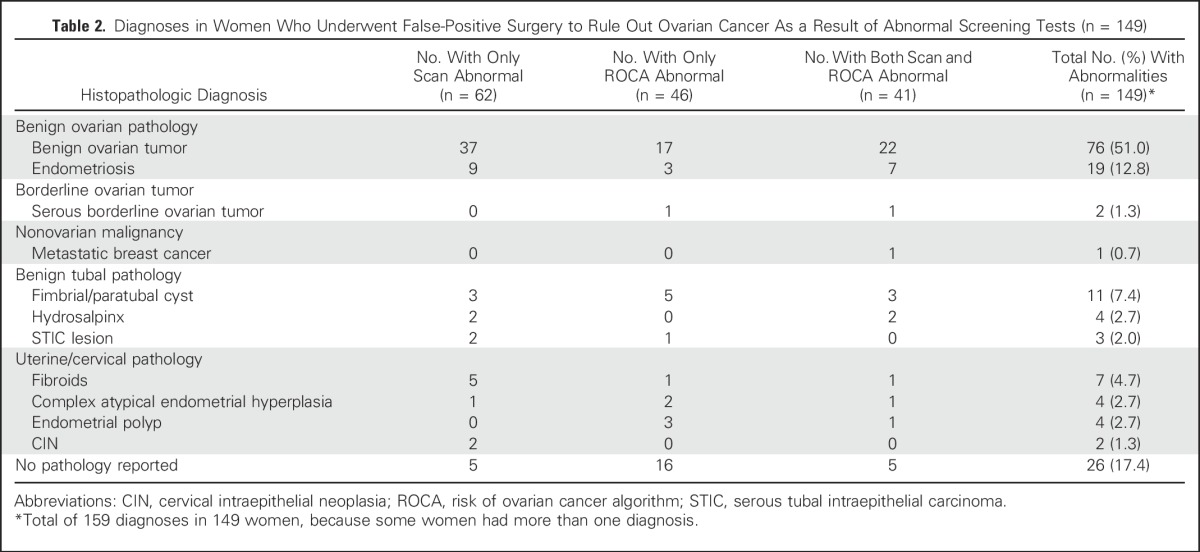
Diagnoses in Women Who Underwent False-Positive Surgery to Rule Out Ovarian Cancer As a Result of Abnormal Screening Tests (n = 149)

### Invasive OC/FTC/PPC

Thirty-seven women were diagnosed with invasive cancer before February 28, 2016 (Table 3); nineteen occurred during 13,728 WSY < 365 days after prior screen and/or withdrawal (annual incidence 0.14%). In addition, 18 women were diagnosed > 365 days after their last UK FOCSS screen (median, 666 days; range, 400 to 2,159 days). The median age at diagnosis in the 37 women diagnosed with OC/FTC/PPC was 50 years (range, 37 to 79 years). All diagnoses occurred in families with hereditary breast-ovarian cancer. Thirty-four (91.2%) of the 37 women were diagnosed with high-grade serous carcinoma. Cancers in 31 (83.8%) of the 37 women occurred in mutation carriers—24 (64.9%) were *BRCA1* carriers and seven (18.9%) were *BRCA2* carriers. Three (8.1%) of the 37 women were *BRCA1/2* negative; one (2.7%) of the 37 women had a *BRCA2* variant of unknown significance; two (5.4%) of the 37 women were untested. Of the 37 women diagnosed with OC/FT/PPC, 23 (62.2%) knew they carried pathogenic mutations and 14 (37.8%) had a history of breast cancer. No OC occurred in women with a family history of LS or those who were mutation carriers for the syndrome (n = 192; 558 WSY).

**Table 3.**
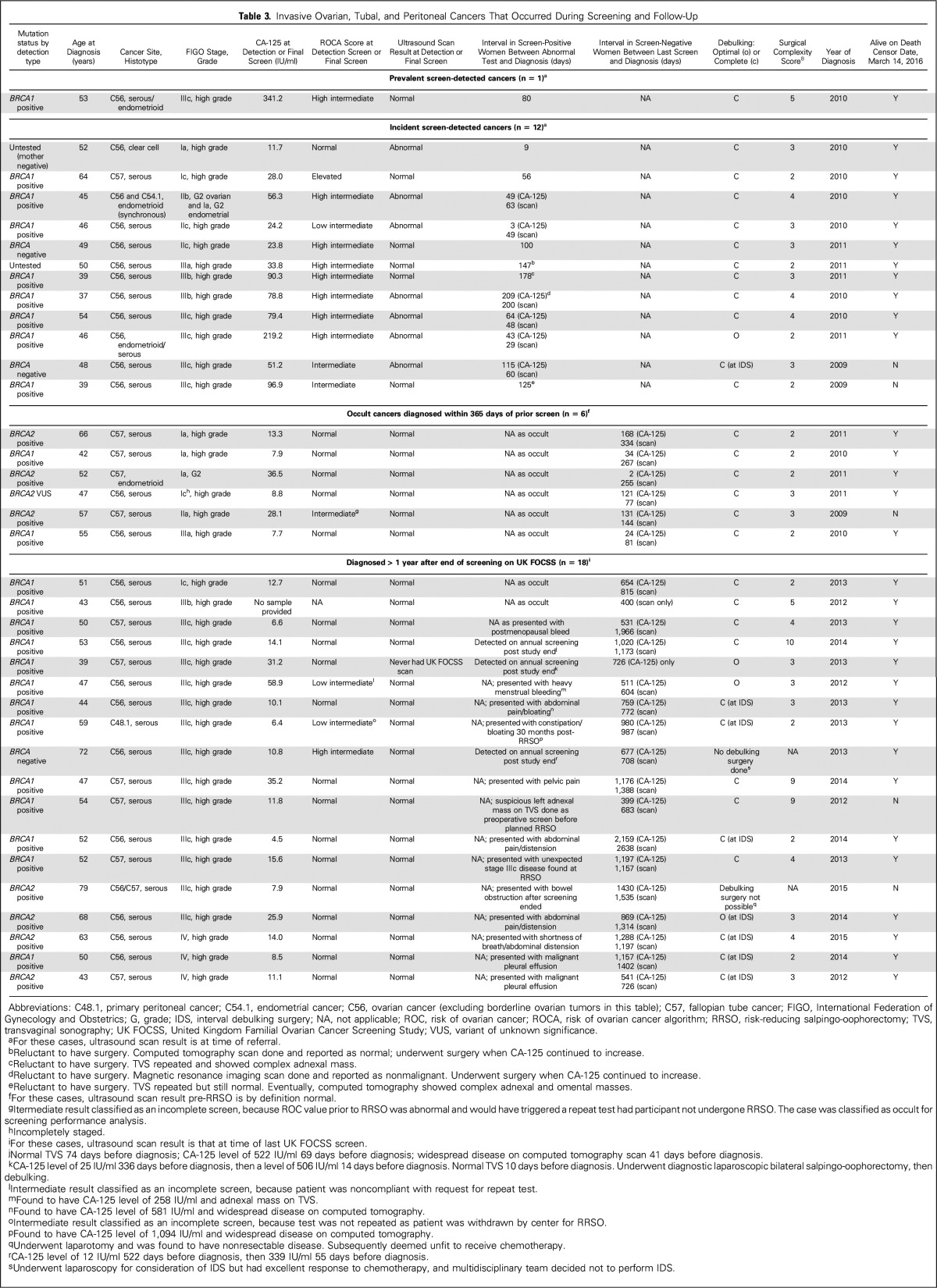
Invasive Ovarian, Tubal, and Peritoneal Cancers That Occurred During Screening and Follow-Up

The 19 invasive OC/FTCs diagnosed within 365 days of prior screen included one prevalent screen-positive OC (International Federation of Gynecology and Obstetrics stage IIIc) and 18 incident cancers. Twelve of the 18 incident OC/FTCs were screen detected and six were occult cancers identified at RRSO. Of the 12 patients with incident screen-detected cancer, 11 (91.7%) had an abnormal ROC and 5 (41.7%) had a normal TVS at detection (compared with zero of 13 patients who had normal TVS at detection in UK FOCSS Phase I; *P* = .015). The median CA-125 level at detection was 53.8 iU/ml (range, 11.7 to 219.2 iU/ml) in UK FOCSS Phase II (< 30 iU/ml in four of 12 patients) compared with 73 iU/ml (range, 4 to 3,874 iU/ml) in Phase I, which did not mandate assay type and recommended premenopausal and postmenopausal cutoffs of 35 and 30 iU/ml, respectively, rather than according to the ROCA. Five (38.5%) of the 13 screen-detected OC/FTCs (CI, 13.9% to 68.4%) and 5 (83.3%) of the six occult OC/FTCs (CI, 35.9% to 99.6%) were stage I to II. Overall, 10 (52.6%) of the 19 cancers diagnosed within 365 days of prior screen were stage I to II (CI, 28.9% to 75.6%).

Eighteen cancers were diagnosed > 365 days after the end of UK FOCSS screening. Two occult cancers were detected at RRSO, three cancers were detected at annual screening performed locally, and 13 were detected when women presented with symptoms. Only one (5.6%) of the 18 cancers was diagnosed at stage I to II (CI, 0.2% to 27.3%).

Women were significantly less likely to be diagnosed with stage IIIb to IV OC during UK FOCSS Phase II screening (seven [36.8%] of 19; CI, 16.3% to 61.6%) compared with those diagnosed subsequently (17 [94.4%] of 18; CI, 72.7% to 99.9%; *P* < .001). Twelve (92.3%) of 13 women who had screen-detected cancers had zero postsurgical residual disease (CI; 64.0% to 99.8%). Overall, 18 (94.8%) of 19 women diagnosed with OC during UK FOCSS had zero postsurgical residual (CI, 74.0% to 99.9%) compared with 13 (72.2%) of 18 women who were diagnosed subsequently (CI, 46.5% to 90.3%; *P* = .09). None of the women diagnosed during UK FOCSS required complex surgery, one had interval surgery. Three of the subsequently diagnosed women required complex surgery, seven had interval surgery, and two had no debulking (Table 3). The proportion of women diagnosed with OC during UK FOCSS who had neoadjuvant chemotherapy (1 [5.3%] of 19 women; CI, 0.1% to 26.0%) was significantly lower than in the women diagnosed subsequently (eight [44.4%] of 18 women; CI, 21.5% to 69.2%; *P* = .008). The mean surgical complexity score^28^ in women diagnosed during screening or less than 365 days after the final screen was 2.7 compared with 4.3 in those diagnosed subsequently (Mann-Whitney *U* test, *P* = .16).

### Screening/Surgical Intervals

The median delay in incident detection screens in this Phase II study was 6 days (range, 0 to 87 days) compared with 88 days (range, 6 to 737 days) in Phase I (gamma generalized linear model, *P* = .004). The median interval between detection screen and diagnosis in this Phase II study was 82 days (range, 9 to 209 days) compared with 79 days (range, 15 to 184 days) in Phase I (*P* = not significant). Reasons for the delay included falsely reassuring scans and reluctance to undergo surgery (Table 3).

### Screening Performance

All 13 cancers (100%) in women who did not undergo RRSO were screen detected (CI, 75.3% to 100%). Hence, for modeled sensitivity, the lower confidence limit of 75.3% was used to conservatively estimate the proportion of occult cancers which would have been screen detected had women not undergone RRSO.

Modeled sensitivity, PPV, and negative predictive value (NPV) for the detection of OC/FTC at 1 year for the whole population were 94.7% (CI, 74.0% to 99.9%), 10.8% (CI, 6.5% to 16.5%), and 100% (CI, 100% to 100%), respectively. PPV was significantly better in *BRCA1/2* carriers than in women who had an unknown mutation status (Table 4).

**Table 4.**
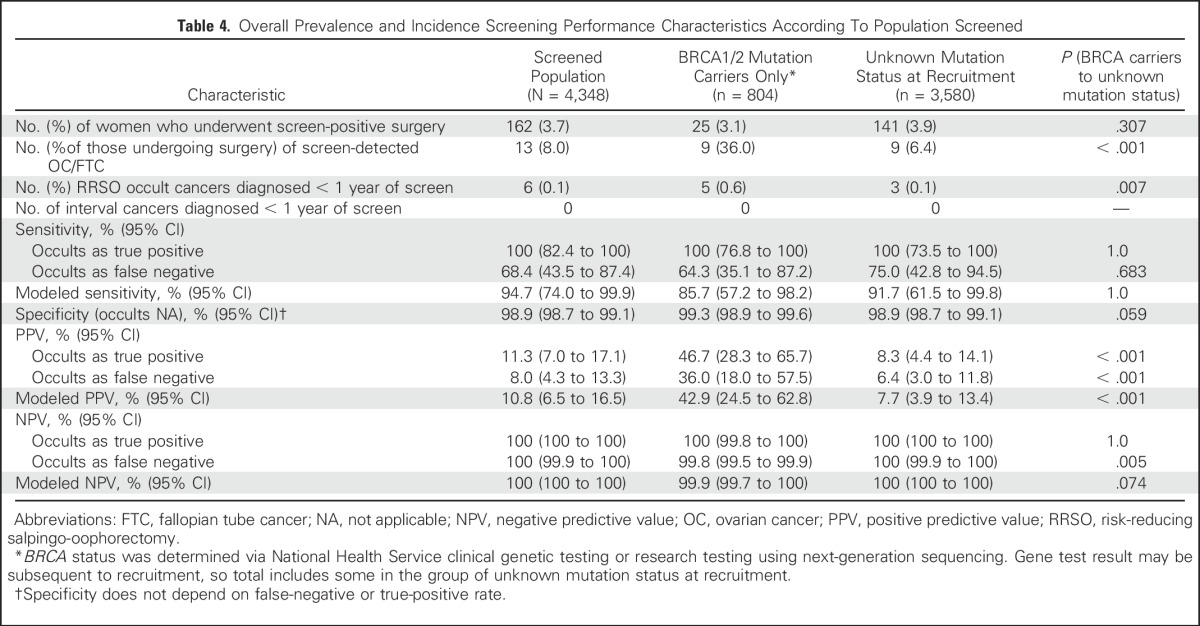
Overall Prevalence and Incidence Screening Performance Characteristics According To Population Screened

### Comparison of Phase I With Phase II

Key comparisons of UK FOCSS Phase I and Phase II are listed in Table 5. Rates of clinically presenting interval cancers, zero residual disease after surgery, modeled sensitivity, proportions of women diagnosed with cancer stage less than IIIb, screening delays, and proportions with normal scans at referral were all better in Phase II, but only the comparisons of screening delays and proportions with normal scans at referral were significant.

**Table 5.**
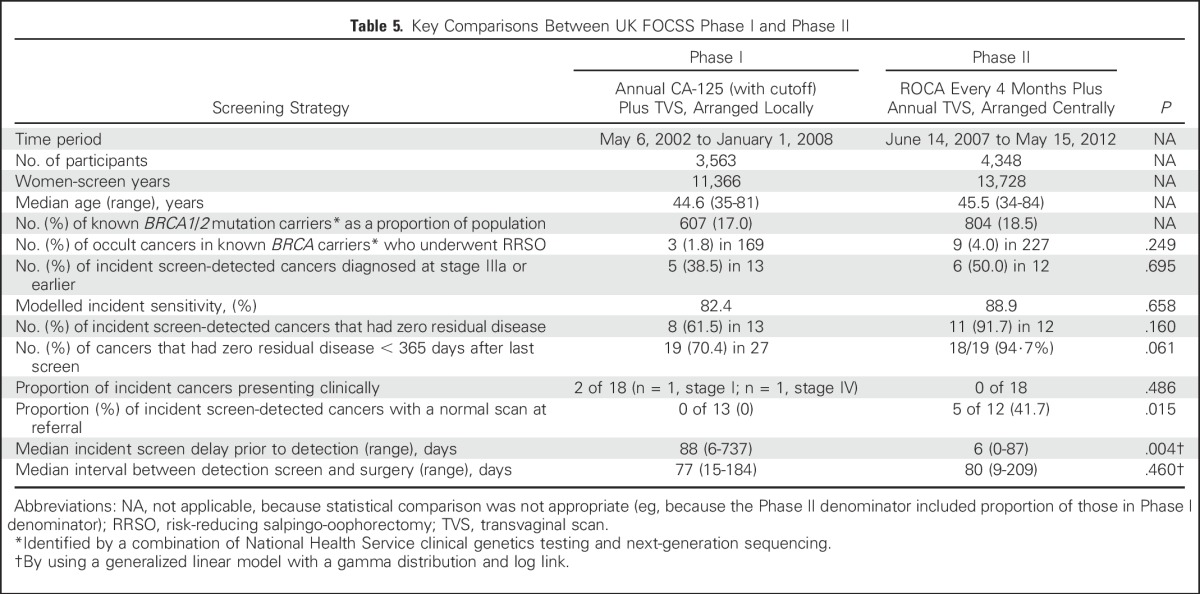
Key Comparisons Between UK FOCSS Phase I and Phase II

## DISCUSSION

These UK FOCSS Phase II results suggest that in a high-risk population, ROCA-based multimodal screening every 4 months, alongside reminders of the effectiveness of RRSO, is associated with high sensitivity, significantly lower high-volume disease, and a high zero residual disease rate after surgery compared with women from the same cohort in whom cancer was diagnosed > 1 year after screening ended. Two similar US studies were published while this paper was in press as a combined analysis, with three of six incident cancers found at stage I/II.^29^

The strengths of this study are its size, multicenter setting, centralized screening with a validated algorithm, and reliable multiple-source follow-up. A limitation is the nonrandomized design. However, data about OC diagnosed after screening ended allowed comparisons in the absence of a nonscreening arm. Other limitations include the unknown mutation status of many participants and the small number of incident cancers, which limited power. Although screening delays were effectively eliminated (median, 6 days), the median interval between abnormal results and surgery continued to be > 2 months (82 days in this Phase II study *v* 79 days in Phase I). There were still some long intervals as a result of patient reluctance to undergo surgery and falsely reassuring imaging associated with an abnormal ROC at referral, as seen in UKCTOCS.^23^

During and within 1 year of UK FOCSS screening, no patients with interval cancers presented with symptoms, sensitivity was high, and there was a significant stage shift compared with patients who had cancers diagnosed more than 1 year after screening ended. The significantly higher proportion of cancers diagnosed at stage IIIa or lower (ie, microscopic abdominal disease at worst) during the study was associated with more primary surgery and with higher zero residual disease achieved with less complex surgery. Published complete cytoreduction rates in clinically presenting *BRCA1/2* carriers ranged from 28% to 30%.^2,30^ The overall findings suggest a screening-mediated reduction in disease volume. It is likely this would translate into reduced surgical morbidity and fewer incomplete resections. It remains unknown whether this would improve survival in screened high-risk women. We were unable to analyze survival, because there were only three deaths in the 37 women with invasive OC/FTC/PPCs at censorship on March 14, 2016. Although this is encouraging, medium-term survival of OC in *BRCA1/2* carriers is better than that of *BRCA1/2*-negative patients.^7,8^

The performance characteristics of screening every 4 months were encouraging; overall incident sensitivity was 94.7%, with occult cancer detection modeled, and PPV was 10.8% (ie, greater than the suggested 10% level for general-population screening).^16^ However, PPV is less relevant in high-risk populations for whom RRSO is already recommended as optimal management. As expected, PPV was better in *BRCA1/2* carriers (42.9%) than in women who had an unknown mutation status (7.7%) because of the lower cancer incidence in women with an unknown status.

The high compliance with blood tests and TVS suggests that the protocol is feasible and acceptable. However, compliance might not be maintained outside a trial. A parallel psychological study found moderate cancer distress at 1 week in women with abnormal ROCA and/or scan results, which led to higher withdrawal from screening.^31^ However, there was no significant effect on general anxiety and/or depression on return to routine screening or at 9 months.

In conclusion, our protocol achieves encouraging performance characteristics, is associated with a low rate of high-volume disease at primary surgery, and had a high zero residual disease rate at low levels of surgical complexity. RRSO remains the treatment of choice for women at high-risk of OC/FTC. In those not ready or willing to undergo surgery, multimodal screening using ROCA every 4 months and TVS (at an interval determined by the ROCA), with regular discussions about the effectiveness of RRSO, appears to be a better option than symptom awareness alone. Such screening should not be viewed as an alternative to surgery, but it does seem to offer a better chance of avoiding a diagnosis of advanced incompletely resectable OC/FTC in the interim.
